# Clinical efficacy of Dupilumab in the treatment of moderate to severe Atopic Dermatitis and Type-II Inflammation-Related comorbidities: A real-world study

**DOI:** 10.12669/pjms.41.9.12369

**Published:** 2025-09

**Authors:** Bo Ding, Yin Lai, Jiahui Zhang, Yanming Lu, Yiwei Wang, Yinshi Guo

**Affiliations:** 1 Bo Ding Department of Pediatrics, Renji Hospital, Shanghai JiaoTong University School of Medicine, Shanghai 201112, P.R. China; 2 Yin Lai Department of Pediatrics, Renji Hospital, Shanghai JiaoTong University School of Medicine, Shanghai 201112, P.R. China; 3 Jiahui Zhang Department of Allergy, Renji Hospital, Shanghai JiaoTong University School of Medicine, Shanghai 201112, P.R. China; 4 Yanming Lu Department of Pediatrics, Renji Hospital, Shanghai JiaoTong University School of Medicine, Shanghai 201112, P.R. China; 5 Yiwei Wang Department of Allergy, Renji Hospital, Shanghai JiaoTong University School of Medicine, Shanghai 201112, P.R. China; 6 Yinshi Guo Department of Allergy, Renji Hospital, Shanghai JiaoTong University School of Medicine, Shanghai 201112, P.R. China

**Keywords:** Dupilumab, Atopic Dermatitis, Type-II Inflammation comorbidities

## Abstract

**Objective:**

To evaluate real-world efficacy and safety of dupilumab in children and adults with moderate to severe atopic dermatitis (AD) combined with Type-II inflammatory diseases.

**Methodology:**

A retrospective analysis was conducted on pediatric and adult AD patients treated with dupilumab at Renji Hospital (July 2021 to November 2023). Changes in medication scores, symptom severity and quality of life (QoL) assessments at three and six months were compared to baseline. Adverse events were monitored.

**Results:**

All 33 patients demonstrated significant improvements in AD medication scores, symptoms and Qol at three/six months vs baseline. Efficacy at three months correlated with six months outcomes. The dupilumab regimen was associated with significantly improved symptoms of Type-II inflammatory comorbidities. Treatment was associated with symptoms that completely disappeared in 18.18% of AR patients and improved in 68.18% of AR cases. Of 11 FA patients, 90.90% reported symptom improvement. The dupilumab regimen led to a 100% and 20% prevalence of symptom improvement in chronic urticaria (CU) and AS patients, respectively, while 80% of AS patients reported complete disappearance of clinical symptoms. Among 15 AC patients, four (26.67%) had symptoms disappeared, seven (46.67%) improved. The dupilumab regimen led to symptom disappearance in one case of a patient with RS. Adverse effects of the treatment were mild.

**Conclusion:**

Dupilumab is an effective and safe treatment for moderate-to-severe AD with Type-II inflammatory comorbidities, significantly improving clinical symptoms, reducing concomitant medication needs and enhancing QoL in real-world settings.

## INTRODUCTION

Atopic dermatitis (AD) is a chronic, prurtic inflammatory skin disease,85% have onset at five years old, about 1/5 of children with AD continue into adulthood with recurring attacks.^1^,^2^ Over 60% of AD patients also have accompanying AF-related comorbidities, as AD is the first step in the allergic process and can subsequently develop into food allergy (FA) (15%), allergic rhinitis (AR) (34%) and bronchial asthma (AS) (20-35%).^1^,^3^ While severe AD is associated with a higher rate of accompanied allergic diseases, the presence of comorbid allergic diseases can, in turn, lead to the worsening of AD in some patients.^4^ These correlations suggest the existence of common underlying immunopathogenic mechanisms and raise the possibility of targeting these mechanisms to treat multiple allergic diseases simultaneously.

Dupilumab is a monoclonal antibody that specifically binds to IL-4Rα, simultaneously inhibits IL-4/IL-13.Phase III clinical trials have shown that dupilumab not only effectively reduces the severity of moderate to severe AD but also has a therapeutic effect on AD-related Type-II inflammatory comorbidities.^5^,^6^ However, the efficacy of dupilumab in the real world, particularly among different populations and their allergic comorbidities, is an area worthy of further exploration.

This study aimed to comprehensively assess changes in clinical scores following dupilumab treatment for moderate-to-severe AD and Type-II inflammatory comorbidities across different populations, to provide guidance for patient-centered personalized treatment strategies.

## METHODOLOGY

Clinical data of 33 children and adults with moderate to severe AD who received dupilumab at Renji Hospital (July 2021 to November 2023) were retrospectively selected.

### Ethical Approval:

This study has been approved by the Ethics Review Committee of Renji Hospital (No.LY2024--63-C Date August 02-2024) and an informed consent form was signed.

### Inclusion criteria:

- Patients aged ≥5 years old, meeting the criteria for moderate to severe AD.^7^
- The Type-II inflammatory diseases meeting the diagnostic criteria of bronchial asthma,^8^ allergic rhinitis,^9^ etc.
- Receive dupilumab treatment for at least six months.

### Exclusion criteria:

- Patients with hypersensitivity to the active ingredient of dupilumab or any other excipients
- Patients who cannot tolerate treatment-related adverse events.
- PIncomplete medical data.
- Patients with other serious skin diseases.

### Dupilumab monoclonal antibody regimen:

All patients were given dupilumab subcutaneously on the upper arm alternately. For patients weighing ≤30kg and ≥60kg, dupilumab administration was done according to the manufacturer’s instructions. For patients weighing 30-60kg, the medication was adjusted to the first dose of 600mg, followed by 300 mg/3 weeks. Among 33 patients in the cohort, 13 also received subcutaneous immunotherapy (SCIT).

### Data collection and efficacy evaluation:

The following data were collected from all patients:

### Demographic and personal information:

age, gender, race and body mass index (BMI=Kg/m²).

### AD-related assessment^10^:

NRS, SCORAD, EASI, IGA, DLQI, POEM, ADCT and medication scores. The scores of AD drugs: one point for antihistamines, three points for strong TCS, two points for medium-effective TCS and one point for weak TCS. After the TCS is reduced, 1.5/1/0.5 points were recorded respectively; TCI 0.1% and 0.03% was scored as two and one, respectively. A small amount of TCI was recorded as 1-0.5 points; Crisaborole ointment received a score of one point; compound Glycyrrhizin Tablets were scored as one point. After the dosage was reduced, 0.5 points were recorded and the cumulative total score was calculated as the overall AD patient medication score.

### Assessment of allergic comorbidities:

(1) AR: 9,11 symptom score, VAS, number of attack days within one month, TMS, RCAT and RQLQ.(2) AS:^8^ ACT and symptom control scores. (3) Urticaria, allergic conjunctivitis and sinusitis: Assessment of improvement, disappearance, or no change.

### FA:

the type of food allergy that improves, disappears, or has no change after the treatmen

### Adverse events:

all treatment-related adverse events.

### Laboratory examination:

total IgE, d1 and mx2.

### Medication history:

### Statistical analysis:

The measurement data were represented by M (Q1, Q3) and the counting data were represented by frequency (%). The χ2 test was used to compare the frequencies of two classification distributions. The Wilcoxon rank sum test was used for comparative analysis of the scores of paired samples. Comparisons between two independent samples are made using the Mann-Whitney U test. P<0.05 indicated statistically significant difference. Spearman correlation analysis assessed relationships between AD efficacy (percentage SCORAD reduction at 3/6 months) and total IgE, age and medication scores.

## RESULTS

As summarized in Table-I, the median age was 11.00 (7.00, 17.00) years. The median BMI was 18.50 (15.40, 23.20) kg/m2 and the median AD disease course was 5.00 (3.50, 9.00) years. As shown in Table-II, treatment with dupilumab was associated with significantly improved medication scores, NRS, SCORAD, EASI, IGA, POEM, DLQI and ADCT scores at three and six-month follow-up compared to the baseline values (P<0.05) in all the patients. At six months after the treatment, the improvement rate of the NRS, SCORAD and EASI scores of pediatric and adult patients were markedly different (P<0.05).

**Table-I:** Baseline patient profile.

	Children	Adults	Total
Age (years)	9.00(6.50, 13.00)	21.00(18.50, 33.00)	11.00(7.00, 17.00)
Gender (Male/Female)	14/11	5/3	19/14
Han ethnic group	24	8	32
Zhuang ethnic group	1	0	1
BMI	16.50(14.80, 20.35)	22.90(20.38, 28.10)	18.50(15.40.23.20)
T-IgE	1042.00(518.00, 1321.00)	3429.00(593.75, 4768.75)	1122.00(562.50, 1873.01)
sIgE≥level 2 (N)	3.00(1.00, 6.00)	10.50(5.25, 14.00)	4.00(2.00, 6.00)
d1	13.00(0.00, 88.50)	79.95(23.18, 98.90)	28.00(1.63, 95.30)
mx2	0.00(0.00, 0.99)	3.18(0.00, 11.68)	0.00(0.00, 3.35)
EOS#	0.77(0.39, 2.14)	0.53(0.31, 0.71)	0.63(0.39, 1.40)
AD course (years)	5.00(3.50, 7.25)	10.00(3.25, 16.00)	5.00(3.50.9.00)
SCORAD score	42.30(35.25.54.60)	46.30(31.00, 49.33)	45.00(35.25, 53.40)

BMI: Body mass index; T-IgE: total IgE; EOS: eosinophils; NRS: Pruritus numeric rate scale;

SCORAD: scoring atopic dermatitis score, EASI: eczema area and severity index,

IGA: Investigator Global Assessment; DLQI: Dermatological Life Quality Index Questionnaire;

POEM: Patient-Oriented Eczema Measure, AD: Atopic Dermatitis; d1: house dust mite; mx2: mixed molds

**Table-II:** Changes in scores of 8 AD indicators before and after treatment in 33 AD patients [M(Q1, Q3).

Categorization	Case number	Medication score	NRS	SCORAD	EASI	IGA	POEM	DLQI	ADCT
Total - baseline	33	3.00(1.00, 3.00)	6.00(6.00, 8.00)	45.00(35.25, 53.40)	7.20(4.20, 13.80)	3.00(3.00, 4.00)	14.00(12.50, 19.00)	13.00(9.00, 20.00)	14.00(8.50, 20.50)
Total - 3 months		1.00*(0.00, 2.00)	2.00*(2.00, 3.00)	20.00*(13.80, 29.95)	1.90*(0.50, 3.85)	2.00*(1.00, 3.00)	4.00*(2.50, 8.00)	6.00*(1.00, 9.50)	5.00*(2.50, 6.00)
Total – 6 months		0.50*(0.00, 1.00)	2.00*(1.00, 3.00)	15.70*(4.70, 20.95)	0.80*(0.15, 2.40)	1.00*(1.00, 2.00)	4.00*(1.00, 6.00)	4.00*(0.50, 7.50)	3.00*(1.00, 6.00)
Children-baseline	25	3.00(2.00, 3.00)	7.00(6.00, 8.00)	42.30(35.25, 54.60)	5.90(4.20, 12.35)	3.00(3.00, 4.00)	16.00(14.00, 20.50)	12.00(7.50, 20.00)	15.00(8.50, 19.00)
Children-3 months		1.00*(0.00, 1.50)	2.00*(2.00, 2.50)	15.90*(10.35, 27.50)	1.40*(0.45, 2.55)	1.00*(1.00, 2.00)	4.00*(2.00, 8.00)	4.00*(1.00, 8.00)	4.00*(2.00, 6.00)
Children-6 months		0.50*(0.00, 1.00)	2.00*(0.00, 2.50)	13.60*(0.50, 20.70)	0.70*(0.00, 1.65)	1.00*(0.50, 2.00)	4.00*(0.50, 6.50)	1.00*(0.50, 6.00)	3.00*(0.00, 6.00)
Adults-baseline	8	1.50(1.00, 2.75)	5.00(3.25, 6.00)	46.30(31.00, 49.33)	8.20(3.45, 19.13)	3.50(3.00, 4.00)	12.00(8.25, 13.75)	13.50(9.75, 22.00)	11.50(8.25, 23.50)
Adults-3 months		1.00(0.13, 2.00)	2.00*(2.00, 3.00)	28.55*(18.50, 35.48)	4.15*(1.33, 10.35)	3.00*(2.25, 3.00)	7.00*(3.25, 9.50)	9.50*(3.50, 10.75)	6.00*(4.50, 8.25)
Adults-6 months		0.25*(0.00, 1.00)	2.50*(1.25, 3.38)	18.40*(11.60, 31.28)	2.00*(0.60, 4.13)	1.50*(1.00, 3.00)	3.50*(2.25, 6.00)	8.00*(1.75, 9.75)	4.00*(2.00, 6.00)
Z1		-4.463	-4.976	-5.012	-5.012	-4.969	-5.016	-4.921	-4.995
P1		0.000	0.000	0.000	0.000	0.000	0.000	0.000	0.000
Z2		-4.020	-4.397	-4.372	-4.372	-4.284	-4.376	-4.261	-4.345
P2		0.000	0.000	0.000	0.000	0.000	0.000	0.000	0.000
Z3		-2.032	-2.106	-2.521	-2.524	-2.598	-2.527	-2.527	-2.527
P3		0.042	0.035	0.012	0.012	0.009	0.012	0.012	0.012
Z4		-0.022	-2.523	-2.570	-2.285	-1.593	-0.697	-0.994	-0.803
P4		0.982	0.012	0.010	0.022	0.111	0.486	0.320	0.422

(* denotes P<0.05 for each indicator at different time points compared to baseline; Z1, P1 denotes comparison of all patients to baseline after 6 months of treatment; Z2, P2 denotes comparison of pediatric patients to baseline after 6 months of treatment; Z3, P3 denotes comparison of adult patients to baseline after 6 months of treatment; Z4 and P4 denote comparison of improvement in each indicator in pediatric and adult patients at 6 months after treatment).NRS: Pruritus numeric rate scale; SCORAD: scoring atopic dermatitis score, EASI: eczema area and severity index, IGA: Investigator Global Assessment; DLQI: Dermatological Life Quality Index Questionnaire; POEM: Patient-Oriented Eczema Measure, ADCT: Atopic Dermatitis Control Tool

**Fig.1:**
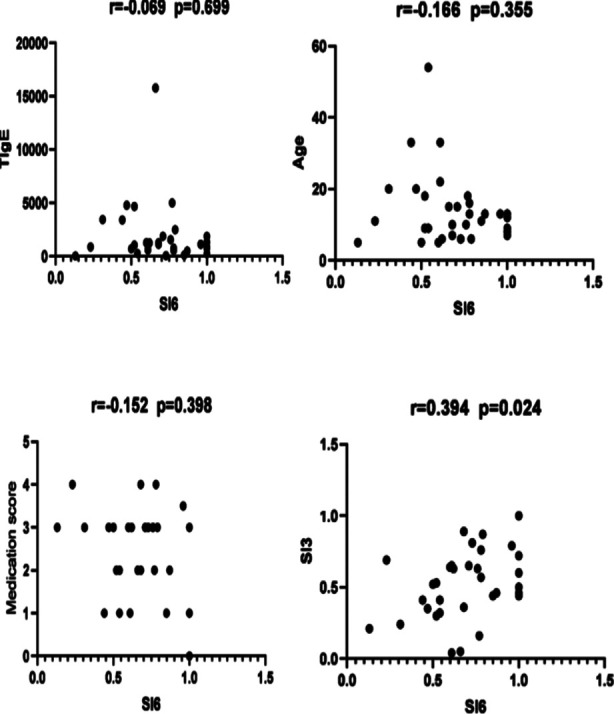
Correlation analysis of AD efficacy.

A Spearman correlation analysis (Fig.1) showed that the AD efficacy after three months of treatment was significantly related to the efficacy of AD treatment at six months (P<0.05). As shown in Table-III, dupilumab treatment resulted in overall improvement of Type-II inflammation-related comorbidities. Of 22 AR patients, 4 (18.18%) reported disappearing symptoms and 14 patients (68.18%) reported symptom improvement. Of 11 FA patients, 10 (90.90%) reported improved symptoms. There were three cases of CU, with all the patients reporting symptom improvement. In five AS patients, symptoms disappeared in 4 (80.00%) and symptom improvement was reported in one patient (20.00%). Of 15 cases of AC, 4 (26.67%) had symptoms disappeared and 7 (46.67%) had symptoms improved. There was one case of RS in the cohort, reporting disappeared symptoms (100%). As shown in Table-IV, the main reported adverse effects in the cohort included upper respiratory tract infections, skin conditions, eye diseases, etc, that were easily controlled (Table-IV).

**Fig.2:**
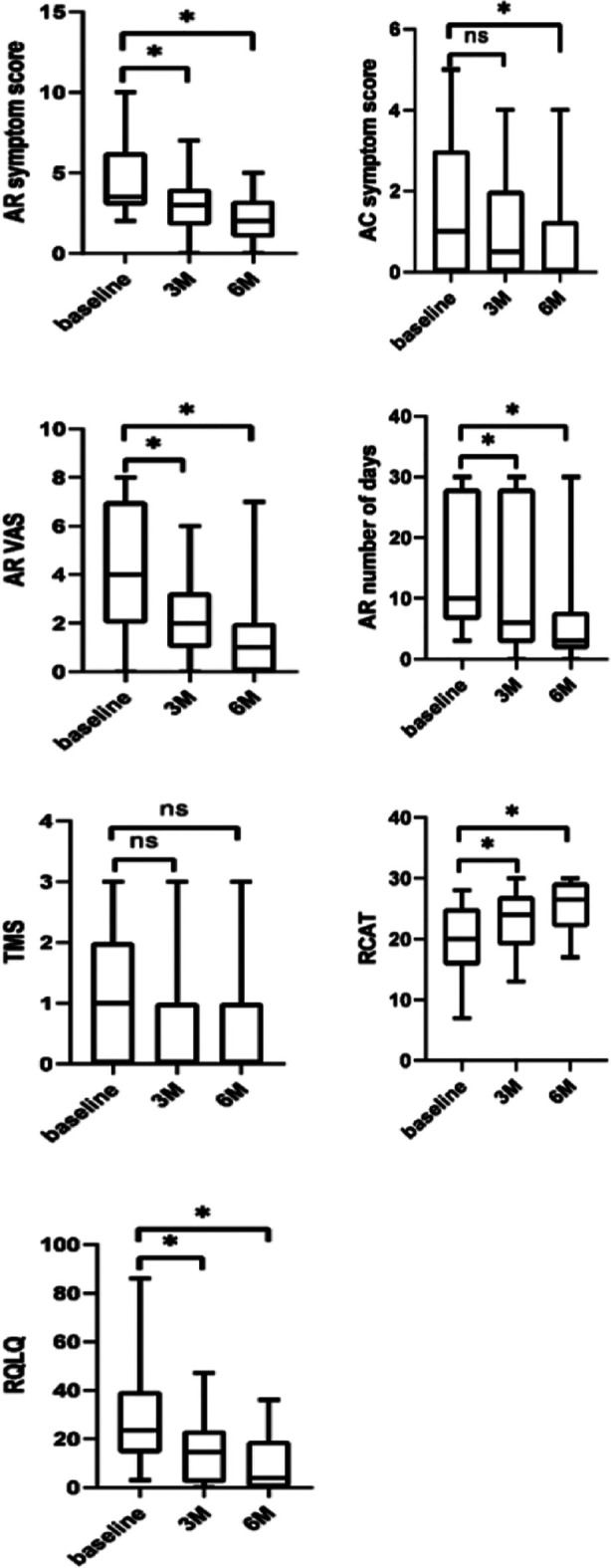
Changes in Type-II inflammatory co-morbidity-nasal-conjunctivitis 6-indicator score before and after treatment in AD patients.

**Table-III:** Remission of Type-II inflammatory co-morbidities in AD patients before and after treatment.

complication	N	Relief before treatment	Disappearance of symptoms (N, %)	Symptomatic improvement (N, %)	Deterioration or no improvement (N, %)
AR	22	1	4(18.18)	14(68.18)	4(18.18)
FA	11	1	0(0.00%)	10(90.90%)	1(9.09%)
CU	3	0	0(0.00%)	3(100%)	0(0.00)
AS	5	3	4(80.00%)	1(20.00%)	0(0.00%)
AC	15	0	4(26.67)	7(46.67)	4(26.67)
RS	1	0	1(100.00%)	0(0.00%)	0(0.00%)

AR: allergic rhinitis; FA: food allergy; CU: chronic urticaria; AS: asthma; AC: allergic conjunctivitis; RS: rhinosinusitis.

**Table-IV:** Adverse effects.

	infectious disease				Skin and soft tissue							eye disease			Injectable local reactions			neuroscience		others
	upper respiratory tract infection	oral herpes (medicine)	otitis media	flu	Erythema of the neck	facial erythema	acroerythema	Transient aggravation of dermatitis	skin infection	herpetic eczema	pustulosis	conjunctivitis	keratitis (inflammation of the cornea)	Itchy eyes	pain	skin flush	pruritus	headache	fatigue	EOS elevated
N	7	2	1	4	5	8	2	6	2	3	1	5	1	8	5	1	1	1	1	1
%	42.42%				81.81%							42.42%			21.21%			3.03%		6.06%
mitigation method	Symptomatic/supportive				Skin infections and herpetic eczema relieved by antibiotic ointment/oral acyclovir; other symptoms relieved by topical TCS							Anti-inflammatory eye drops/observation			observation			saridontetracycline (medicine)		Rest/observation

**Supplementary Table-I:** Changes in Type-II inflammatory co-morbidity-nasal-conjunctivitis 6-indicator scores before and after treatment in patients with AD.

	AR symptom score	AC Symptom Score	VAS	Number of days of seizures in 1 month	RCAT	RQLQ	TMS
baseline	3.50(3.00, 6.25)	1.00(0.00, 3.00)	4.00(2.00, 7.00)	10.0(6.50, 28.00)	20.00(15.75, 25.00)	23.50(14.25, 39.50)	1.00(0.00, 2.00)
3 months	3.00(1.75, 4.00)	0.50(0.00, 2.00)	2.00(1.00, 3.25)	6.00(2.75, 28.00)	24.00(19.00, 27.00)	14.50(2.00, 23.25)	0.00(0.00, 1.00)
6 months	2.00(1.00, 3.25)	0.00(0.00, 1.25)	1.00(0.00, 2.00)	3.00(1.75, 7.75)	26.50(22.00, 29.25)	4.00(0.00, 19.00)	0.00(0.00, 1.00)
Z1	-2.685	-1.754	-2.997	-2.446	-3.983	-3.118	-1.691
P1	0.007	0.079	0.003	0.014	0.000	0.002	0.091
Z2	-3.526	-2.086	-3.786	-3.647	-4.023	-4.109	-1.821
P2	0.000	0.037	0.000	0.000	0.000	0.000	0.069

Z1, P1 represents comparison with baseline after 3 months of treatment for all AR patients; Z2, P2 represents comparison with baseline after 6 months of treatment for all AR patients); AR: allergic rhinitis; FA: food allergy; VAS: visual analog scale; RCAT: Rhinitis Control Assessment Test; RQLQ: Rhinoconjunctivitis Quality of Life Questionnaire; TMS: Total medication score.

**Supplementary Table-II:** Improvement in Type-II inflammatory co-morbidity-food allergy in AD patients before and after treatment.

Cases/ Food	Milk	Egg	Barley	Watermelon	Dragon fruit	Tomato	Fish	Nuts	Mango	Seafood	Chicken	Chilli
Case 1	improved	improved	improved									
Case 2	improved	improved								maintained		
Case 4		maintained		improved	improved	improved	maintained					
Case 5							improved					
Case 6	improved							improved				
Case 12									alleviated	improved		
Case 23										improved		
Case 24	untried		alleviated								untried	
Case 26		improved					improved				improved	improved
Case 27										improved		

**Supplementary Table-III:** Improvement in Type-II inflammatory co-morbidity-AS in AD patients before and after treatment.

	AS symptom score	VAS	Number of asthma attack days in 1 month	ACT control score	TMS
baseline	4.00(0.50, 5.00)	4.00(0.50, 5.00)	3.00(1.50, 17.00)	21.00(18.00, 24.00)	2.00(1.00, 2.50)
3 months	1.00(0.00, 3.00)	2.00(0.00, 3.00)	1.00(0.00, 6.50)	25.00(22.50, 25.50)	2.00(1.00, 2.50)
6 months	0.00(0.00, 0.50)	0.00(0.00, 0.00)	0.00(0.00, 0.00)	25.00(24.00, 26.50)	2.00(0.00, 2.00)
Z1	-1.604	-1.890	-1.826	-1.841	0.000
P1	0.109	0.059	0.068	0.066	1.000
Z2	-1.841	-1.841	-1.841	-1.841	-1.000
P2	0.066	0.066	0.066	0.066	0.317

(Z1, P1 represents comparison with baseline after 3 months of treatment for all AS patients; Z2, P2 represents comparison with baseline after 6 months of treatment for all AS patients); ACT: Asthma Control Tool; TMS: Total medication score.

## DISCUSSION

This retrospective study of 33 moderate-to-severe AD patients demonstrated significant improves in NRS, SCORAD and EASI at three/six months with dupilumab (all p<0.05). Koskeridis^12^ conducted a systematic review and meta-analysis of a total of 12 adult (n=3817) and two children/adolescent (n=618) placebo-controlled randomized clinical trials and showed that dupilumab treatment reduced EASI, SCORAD, NRS and IGA scores in both children/adolescents and adults with comparable effectiveness. This is consistent with our results. We showed that six months after the treatment, the treatment effect in children/adolescents was somewhat better than in the adult population, as indicated by NRS, SCORAD and EASI scores (P<0.05).

A possible explanation for this observed effect is that although Th2-type inflammation is the main pathogenesis of AD, the specific pathways differ at different ages. For instance, the Th22, Th17 and Th1 pathways are activated in adults.^13^ Age-related decreases in immune barrier function and changes in immune response may be also related to different forms of AD and its manifestations.^2^ Our study’s AD efficacy correlation analysis showed that the improvement of SCORAD at six months after the treatment was only significantly associated with the improvement of SCORAS three months after the treatment. Such results may be related to the small sample size of the adult cohort in this study. This observation is more consistent with the real-life clinical situation. However, it is important to mention that comparing adult and pediatric data in our study should be taken cautiously due to the multiple potential age-related confounding factors that were not accounted for. Further studies in pediatric and adult populations are needed to validate our observations.

Our report found that dupilumab significantly improved Type-II inflammation-related comorbidities. A randomized, double-blind, controlled trial of adult patients with AS and perennial allergic rhinitis (PAR) showed that 24 weeks of treatment with dupilumab (300 mg q2w) significantly improved AR-related symptom scores compared with the placebo group.^14^ Another study of 41 patients with asthma and rhinitis who were still uncontrollable after basic drug treatment showed that six-months treatment with SQ House Dust Mite (HDM) Sublingual Immunotherapy (SLIT) combined with dupilumab improved ACQ-5 (P < 0.05), AQLQ (P < 0.05) and RQLQ (P < 0.05) scores and lung function of patients.^15^

In our study, RCAT control scores and RQLQ of 22 AR patients were significantly improved six months after the treatment compared with the baseline (P < 0.05). No significant improvement in the medication score was observed. We may speculate that the severity of AR in children is mainly mild, intermittent and requires less medication. In contrast, adult AR patients are more likely to have moderate to severe symptoms and require continuous medication. However, as the medication effects are usually poor, patients discontinue them. Four AR patients (two children and two adults) in our cohort responded poorly to the treatment regimen. It is plausible that in two pediatric AR patients, poor response may be related to a large number of grade ≥2 allergens, high sensitization values and various influencing factors (seasonal changes). In two adult patients, the poor response may be related to a long course of severe rhinitis. There were only five AS patients in our cohort. One patient with asthma since childhood presented with declined lung function that showed marked improvement after the treatment. Asthma-related symptoms of other patients completely disappeared and their ACT control scores significantly improved.

AD is a risk factor for food allergy.^16^ Allergens pass through the damaged skin barrier, activating and producing downstream cytokines, including IL-4 and IL-13. If targeted therapy is used early in life to help establish immune tolerance in the gastrointestinal mucosa, the development of allergic diseases can theoretically be prevented.^17^ A study by Spekhorst et al.^18^ showed that dupilumab treatment can induce a sustained decrease in sIgE levels (a one-year treatment cycle, a decrease of 50%-60%; a three-year treatment cycle, a decrease of approximately 80%) and in the severity of allergy symptoms on accidental ingestion of allergic foods. Case reports documented increased tolerance to foods that had previously caused allergic reactions after using dupilumab.^19^ A total of 10 patients in our study had food allergies (allergic reactions after eating a certain food). During the treatment of AD, food tolerance in patients with allergic reactions increased, which is consistent with the case reports.

Our results show that while dupilumab can relieve symptoms of conjunctivitis, it may also induce ocular surface diseases (mild to severe conjunctivitis, blepharitis, keratitis, etc.). Phase 2b and three clinical trial results show that the incidence of conjunctivitis in patients treated with Dupilumab dupilumab is 8% and the pathogenesis of this effect is still unclear.^20^ Possible mechanisms may involve higher levels of atopic keratoconjunctivitis (AKC) ligand, increased eosinophil counts in tear fluid, or inhibition of IL-13.^21^ The most common ocular surface disease, secondary to dupilumab, is usually mild conjunctivitis that may require referral to an ophthalmology specialist in severe cases.^22^

Other reported adverse events of dupilumab include infectious diseases, skin and soft tissue and others. There were no serious complications reported in our study.

### Limitations:

This is a retrospective single-center study with a small sample size, which may affect the robustness and generalizability of our results. A small number of adult patients in our study did not allow us to perform a more in-depth assessment of the age-related differential efficacy of dupilumab. Additionally, there was a variability in the timing of SCIT therapy among study participants, that may have impacted the observed effect of the treatment. Further well-controlled large prospective studies with placebo groups are needed to validate our results.

## CONCLUSION

This study comprehensively evaluated the changes in clinical scores of dupilumab on all aspects of various Type-II inflammatory diseases in AD patients. It compared the real-life efficacy of the treatment in adult and pediatric AD patients. Our results show that dupilumab treatment is safe and has significant overall efficacy. The treatment effect of children with AD is better than that of adults to a certain extent. Our study may be used to update current treatment guidelines and provide AD patients with more personalized treatment approaches.

### Authors’ contributions:

**BD, YL, JZ and YL:** Literature search, study design and manuscript writing.

**YW and YG:** Were involved in data collection, data analysis and interpretation. Critical Review.

**BD, YL, JZ and YL:** Manuscript revision and validation and is responsible for the integrity of the study.

All authors have read and approved the final manuscript.
